# Variables Influencing the Effectiveness of Creatine Supplementation as a Therapeutic Intervention for Sarcopenia

**DOI:** 10.3389/fnut.2019.00124

**Published:** 2019-08-09

**Authors:** Darren G. Candow, Scott C. Forbes, Philip D. Chilibeck, Stephen M. Cornish, Jose Antonio, Richard B. Kreider

**Affiliations:** ^1^Faculty of Kinesiology and Health Studies, University of Regina, Regina, SK, Canada; ^2^Department of Physical Education, Brandon University, Brandon, MB, Canada; ^3^College of Kinesiology, University of Saskatchewan, Saskatoon, SK, Canada; ^4^Faculty of Kinesiology and Recreation Management, University of Manitoba, Winnipeg, MB, Canada; ^5^Department of Health and Human Performance, Nova Southeastern University, Davie, FL, United States; ^6^Department of Health and Kinesiology, Texas A&M University, College Station, TX, United States

**Keywords:** muscle, strength, resistance training, mechanisms, safety

## Abstract

Sarcopenia is an age-related muscle condition characterized by a reduction in muscle quantity, force generating capacity and physical performance. Sarcopenia occurs in 8–13% of adults ≥ 60 years of age and can lead to disability, frailty, and various other diseases. Over the past few decades, several leading research groups have focused their efforts on developing strategies and recommendations for attenuating sarcopenia. One potential nutritional intervention for sarcopenia is creatine supplementation. However, research is inconsistent regarding the effectiveness of creatine on aging muscle. The purpose of this perspective paper is to: (1) propose possible reasons for the inconsistent responsiveness to creatine in aging adults, (2) discuss the potential mechanistic actions of creatine on muscle biology, (3) determine whether the timing of creatine supplementation influences aging muscle, (4) evaluate the evidence investigating the effects of creatine with other compounds (protein, conjugated linoleic acid) in aging adults, and (5) provide insight regarding the safety of creatine for aging adults.

## Introduction

The original criteria for determining sarcopenia focused on muscle quantity (1); however, over the past few decades, numerous groups (International Working Group on Sarcopenia, Special Interest Group, European Working Group on Sarcopenia in Older People, Foundation for the National Institutes of Health, Asian Working Group on Sarcopenia, European Society of Clinical Nutrition and Metabolism, and International Sarcopenia Initiative) have expanded this criterion to include muscle strength and physical performance measures (2). Sarcopenia, now identified with an ICD-10-CM code by the World Health Organization (3) occurs in 8–13% of adults ≥ 60 years of age (4). The European Working Group on Sarcopenia in Older People classifies aging adults with low muscle strength (Grip strength test: <27 kg for males, <16 for females; Chair stand test: >15 s for five stands) as being pre-sarcopenic; those with low muscle strength and low muscle quantity (Appendicular skeletal muscle mass: <20 kg for males, <15 kg for females; Appendicular skeletal muscle mass/height^2^: <7.0 kg/m^2^ for males, <6.0 kg/m^2^ for females) as being sarcopenic, and those with low muscle strength, low muscle quantity and low physical performance (Gait speed: ≤0.8 m/s; Short Physical Performance Battery test: ≤ 8 point score; Timed Up-and Go test: ≥20 s; 400 m walk test: non-completion or ≥6 min for completion) as having severe sarcopenia (5). Sarcopenia is associated with disability, frailty, cachexia, morbidity, and various diseases (6, 7). Contributing factors of sarcopenia include changes in neuromuscular function, skeletal muscle morphology, protein kinetics, hormonal regulation, oxidative and inflammatory stress, physical inactivity, and nutrition [for reviews see (8, 9)].

The European Society for Clinical and Economic Aspects of Osteoporosis, Osteoarthritis and Musculoskeletal Diseases [ESCEO; (10)] and International Conference on Sarcopenia and Frailty Research task force (11) recommends that nutritional interventions be emphasized to help overcome sarcopenia. We propose that creatine supplementation has the potential to be an anti-sarcopenic intervention. Three meta-analyses have been performed involving creatine supplementation and resistance training. In the first meta-analysis, Candow et al. (12) found a greater effect from creatine supplementation during resistance training on muscle mass (+0.94 kg) and upper-body maximal strength compared to placebo during resistance training in over 300 participants (>50 years of age). Expanding on these findings, Devries and Phillips (13) showed that creatine supplementation during resistance training resulted in greater gains in muscle mass (+1.33 kg) and upper- and lower-body maximal strength, and physical performance (30-s chair stand test) compared to placebo during training in over 200 aging adults (>45 years of age). In the most recent meta-analysis, Chilibeck et al. (14) showed that creatine supplementation during resistance training significantly increased muscle mass (+1.37 kg), and upper- and lower-body maximal strength compared to placebo during resistance training in over 700 aging adults (>57 years of age). Collectively, these meta-analyses indicate that the addition of creatine to resistance training significantly increases muscle mass (1.21 kg), maximum strength and has promise for improving tasks of physical performance in aging adults. However, variability in the responsiveness to creatine supplementation is typically high in aging adults and several factors determine whether an individual experiences greater gains in muscle mass and muscle/physical performance from creatine supplementation and resistance training. Therefore, the purpose of this perspective paper is to: (1) propose possible reasons for the inconsistent responsiveness to creatine in aging adults, (2) discuss the potential mechanistic actions of creatine on muscle biology, (3) determine whether the timing of creatine supplementation influences aging muscle, (4) evaluate the evidence investigating the effects of creatine with other compounds (protein, conjugated linoleic acid) in aging adults, and (5) provide insight regarding the safety of creatine for aging adults.

### Creatine

**Figure 1:**
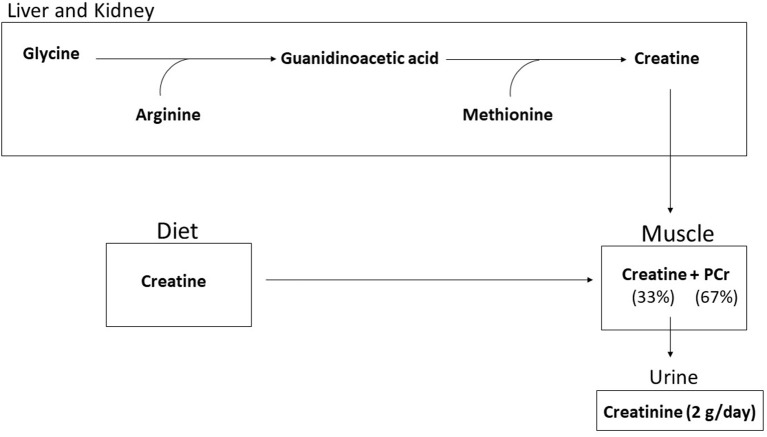
Creatine is synthesized endogenously by a two step process from glycine, arginine, and methoione or through dietary intake. Ninety-five percent of creatine is taken up into the muscle and stored as free creatine (33%) or as phosphorylcreatine (67%). Approximately 2 g per day is broken down to creatinine and excreted.

Creatine (methylguanidine-acetic acid) is a naturally occurring nitrogenous organic acid (15). Ninety-five percent of creatine is stored in skeletal muscle while the remainder is found in brain, liver, kidneys, and testes (16). In skeletal muscle, approximately two-thirds of creatine is bound to phosphate and stored as phosphocreatine (PCr), the remaining one-third of creatine is unbound and stored as free creatine (15). An average 70-kg individual maintains a total creatine pool (PCr + free Cr = total creatine) of ~120 mmol/kg of dry muscle mass (17). Approximately 2 g/day (1–2%) of intramuscular creatine stores are broken down and excreted in the urine as creatinine (15). Both exogenous dietary intake and endogenous *de novo* synthesis are used to replace lost creatine. Dietary sources of creatine include meat, fish, and poultry with trace amounts in plants (18, 19). For example, one pound of uncooked salmon and beef contains about 2 g of creatine (18). Since plants only contain trace amounts, strict vegetarians and vegans typically have lower skeletal muscle total creatine stores (20). For most individuals, ~1–3 g/d of exogenous creatine intake are required to maintain creatine stores depending on total muscle mass and physical activity levels (17, 18, 21). Endogenous *de novo* creatine synthesis occurs in the liver and kidney (15, 18) via a two-step process. First, arginine and glycine form ornithine and guanidinoacetic acid (GAA) by the enzyme arginine glycine amidinotransferase (AGAT). Second, creatine is formed by the transfer of the methyl group from S-adenosyl methionine to guanidinoacetate (22), as shown in Figure 1.

### Creatine Supplementation During Resistance Training in Aging Adults

There is a growing body of literature examining the effects of creatine supplementation and resistance training in aging adults (Table 1). Individual studies involving aging males or aging males and females combined show mixed results. However, studies only involving aging females show more consistent results. This section will summarize findings across studies and discuss possible methodological reasons for the conflicting results. Furthermore, variables influencing the responsiveness to creatine supplementation are also proposed.

**Table 1:** Resistance training and creatine studies in aging adults on performance and body composition changes.

**References**	**Population**	**Supplement dose**	**Resistance training**	**Duration**	**Outcomes**
**WOMEN ONLY STUDIES**					
Alves et al. (23)	*N* = 47; healthy women, Mean age = 66.8 years (range: 60–80 years)	CR (20 g/day for 5 days, followed by 5 g/day thereafter) or PLA with and without RT	RT = 2 days/week	24 weeks	↔ 1 RM strength compared to RT + PLA
Aguiar et al. (24)	*N* = 18; healthy women; Mean age = 65 years	CR (5 g/day) or PLA	RT = 3 days/week	12 weeks	CR ↑ gains in fat-free mass (+3.2%), muscle mass (+2.8%), 1 RM bench press, knee extension, and biceps curl compared to PLA
Chilibeck et al. (25)	*N* = 33; healthy women; Mean age = 57 years	CR (0.1 g/kg/day) or PLA	RT = 3 days/week	52 weeks	↔ lean tissue mass and muscle thickness gains between groups. ↑ relative bench press strength compared to PLA
Gualano et al. (26)	*N* = 30; “vulnerable” women; Mean age = 65.4 years	CR (20 g/day for 5 days; 5 g/day thereafter) or PLA with and without RT	RT = 2 days/week	24 weeks	CR + RT ↑ gains in 1 RM bench press and appendicular lean mass compared to PLA + RT
Neves et al. (27)	*N* = 24 (postmenopausal women with Knee osteoarthritis); Age = 55–65 years	CR (20 g/day for 1 week, followed by 5 g/day) or PLA	RT = 3 days/week	12 weeks	CR ↑ gains in limb lean mass. ↔ 1 RM leg press
**MEN ONLY STUDIES**					
Bemben et al. (28) and Eliot et al. (29)	*N* = 42; healthy men; age = 48–72 years	CR (5 g/day), protein (35 g/day), CR+ protein, or PLA	RT = 3 days/week	14 weeks	↔ lean tissue mass, 1 RM strength
Candow et al. (30)	*N* = 35; healthy men; age = 59–77 years	CR (0.1 g/kg/day) or CR + protein (0.3 g/kg/day) or PLA	RT = 3 days/week	10 weeks	CR and CR + protein conditions combined ↑muscle thickness compared to PLA. CR ↑1 RM bench press ↔ 1 RM leg press
Chrusch et al. (31)	*N* = 30; healthy men; age = 60–84 years	CR (0.3 g/kg/d for 5 days followed by 0.07 g/kg/day) or PLA	RT = 3 days/week	12 weeks	CR ↑ gains in lean tissue mass. CR ↑1 RM leg press, 1 RM knee extension, leg press endurance, and knee extension endurance. ↔ 1 RM bench press or bench press endurance.
Cooke et al. (32)	*N* = 20; healthy men; age = 55–70 years	CR (20 g/day for 7 days then 0.1 g/kg/day on training days)	RT = 3 days/week	12 weeks	↔ lean tissue mass, 1 RM bench press, 1 RM leg press
Eijnde et al. (33)	*N* = 46; healthy men; age = 55–75 years	CR (5 g/day) or PLA	Cardiorespiratory + RT = 2–3 days/week	26 weeks	↔ lean tissue mass or isometric maximal strength
Villanueva et al. (34)	*N* = 14; healthy men; age = 68.7 years	CR (0.3 g/kg/day for 5 days followed by 0.07 g/kg/day) + 35 g protein or PLA	RT = 3 days/week	12 weeks	↔ lean tissue mass or 1 RM bench press
**MEN AND WOMEN STUDIES**					
Bermon et al. (35)	*N* = 32 (16 men, 16 women); healthy; age = 67–80 years	CR (20 g/day for 5 days followed by 3 g/day) or PLA	RT = 3 days/week	7.4 weeks (52 days)	↔ lower limb muscular volume, 1-, 12-repetitions maxima, and the isometric intermittent endurance
Brose et al. (36)	*N* = 28 (15 men, 13 women); healthy; age: men = 68.7, women = 70.8 years	CR (5 g/day) or PLA	RT = 3 days/week	14 weeks	CR ↑ gains in lean tissue mass and isometric knee extension strength; ↔ type 1, 2a, 2x muscle fiber area
Candow et al. (37)	*N* = 39 (17 men, 22 women); healthy; age = 50–71 years	CR (0.1 g/kg) before RT, CR (0.1 g/kg) after RT, or PLA	RT = 3 days/week	32 weeks	CR after RT ↑ lean tissue mass, 1 RM leg press, 1 RM chest press compared to PLA
Collins et al. (38)	*N* = 16 (frail men and women); age = 70 years	CR (4 g/day) and protein (20 g/day) or protein	RT = 2 days/week	14 weeks	↔ lean tissue mass or muscle function
Deacon et al. (39)	*N* = 80 (50 men, 30 women); COPD; age = 68.2 years	CR (22 g/day for 5 day followed by 3.76 g/day) or PLA	RT = 3 days/week	7 weeks	↔ lean tissue mass or muscle strength
Gualano et al. (40)	*N* = 25 (9 men, 16 women); type 2 diabetes; age = 57 years	CR (5 g/day) or PLA	RT = 3 days/week	12 weeks	↔ lean tissue mass
Johannsmeyer et al. (41)	*N* = 31 (17 men, 14 women); healthy; age = 58 years	CR (0.1 g/kg/day) or PLA	RT = 3 days/week	12 weeks	CR ↑ gains in lean tissue mass and 1 RM strength in men only
Pinto et al. (42)	*N* = 27 (men and women); healthy; age = 60–80 years	CR (5 g/day) or PLA	RT = 3 days/week	12 weeks	CR ↑ gains in lean tissue mass. ↔ 10 RM bench press or leg press strength
Tarnopolsky et al. (43)	*N* = 39 (19 men, 20 women); healthy; age = 65–85 years	CR (5 g/day) + CLA (6 g/day) or PLA	RT = 2 days/week	26 weeks	CR + CLA ↑ gains in lean tissue mass, muscular endurance, isokinetic knee extension strength

*CR, creatine; PLA, placebo; RT, resistance training; CLA, conjugated linoleic acid; RM, repetition maximum*.

While it is difficult to compare results across studies in aging males, differences in training methodologies may be involved. In the Candow et al. (30) and Chrusch et al. (31) studies (both showing a positive effect from creatine), participants were directly supervised during each training session. However, in the Cooke et al. (32) study, participants were only supervised during weeks 1, 2, 6, 8, and 11. Supervised resistance training leads to greater muscle benefits compared to unsupervised training (44). Furthermore, the sample sizes were larger in the Candow et al. (30) and Chrusch et al. (31) studies which increased statistical power compared to the Cooke et al. (32) study. The study by Eijnde et al. (33) incorporated both resistance and aerobic exercise components into the training intervention which introduces the possibility of muscle interference [i.e., blunting of muscle growth and performance when performing both resistance-training and aerobic exercise in the same training program; (45)]. In addition, the resistance training protocol focused on developing muscular endurance (20–30 repetition range) rather than muscular strength, a primary dependent measure assessed.

Five studies have investigated the effects of creatine supplementation during resistance training in aging females, as shown in Table 1. In postmenopausal osteopenic or osteoporotic females, Gualano et al. (26) showed that creatine supplementation during supervised resistance training produced greater gains (relative) in appendicular lean tissue mass (assessed by DXA) and upper-body (bench press) strength compared to placebo during resistance training. Previous work by Neves et al. (27) also found a beneficial effect from creatine on lower-limb lean tissue mass and indices of physical performance (timed-stand test) in postmenopausal women with knee osteoarthritis compared to placebo; however, there was no effect of creatine supplementation on total-body lean mass or muscle strength when compared to placebo. Additional work in postmenopausal women showed that creatine supplementation increased lean tissue mass, strength (bench press, knee extension, biceps curl), and tasks of physical performance (30-s chair stand, arm curl test, lying prone-to-stand test) compared to placebo (24). Chilibeck et al. (25) showed that postmenopausal females who ingested creatine daily during supervised whole-body resistance training experienced greater gains in relative upper-body maximal strength (bench press) compared to females on placebo. Finally, postmenopausal women who ingested creatine during a supervised strength training program had greater gains in lower-body (leg press) strength compared to females who ingested creatine or placebo but did not strength train. There were no differences between females who consumed creatine or placebo during training (23). Collectively, these results suggest that creatine supplementation during supervised resistance training is an effective lifestyle intervention for improving muscle mass and muscle/physical performance in aging postmenopausal women.

Research is limited regarding the effectiveness of creatine supplementation during resistance training in aging males and females combined, and therefore, there are only a few direct comparisons between males and females for responsiveness to creatine, as shown in Table 1. Brose et al. (36) showed that creatine supplementation increased lean tissue mass and isometric knee extension strength in aging adults compared to placebo. Males on creatine increased ankle-dorsiflexion isometric strength more than females on creatine. Candow et al. (37) and Pinto (42) found increases in strength and lean tissue mass with creatine and resistance training compared to placebo; however, no sex differences were found. Johannsmeyer et al. (41) showed that creatine supplementation increased whole-body lean tissue mass compared to placebo. Males on creatine increased upper-body strength (lat pull-down) and decreased urinary 3-methylhistidine excretion (indicator or whole-body protein catabolism) more than females on creatine. In contrast to these positive studies showing a beneficial effect from creatine, Bermon et al. (35) found no effect on strength or lean tissue mass. This study was limited by a small sample size (*n* = 8 per group) and a shorter training intervention (52 days) compared to the studies showing a positive effect from creatine (36, 37, 41, 42), and had participants perform a limited amount of work (3 exercises performed, 3 sets of 8 repetitions at 80% 1-RM). In two studies investigating disease populations, Deacon et al. (39) found no effect from creatine supplementation during 7 weeks of aerobic and resistance training on changes in muscle mass or performance in aging adults with chronic obstructive pulmonary disease (COPD) compared to those on placebo. In aging adults with type II diabetes, Gualano et al. (40) found no effect from creatine supplementation during 12 weeks of supervised aerobic and resistance combined training on muscle mass or strength compared to those on placebo. Both these disease state studies incorporated aerobic and resistance training into the exercise intervention which may have introduced the muscle interference effect (45). It is important to note that no sex analyses were performed in the studies of Bermon et al. (35), Deacon et al. (39), and Gualano et al. (40).

### Methodological Differences Between Studies

Collectively, inconsistent results across studies (independent of sex), may be related to differences in the exercise training intervention (i.e., supervision vs. non-supervision; duration and volume of training; combination of aerobic and resistance training), health status and sample size.

Furthermore, variables which influence an individual's responsiveness to creatine supplementation should also be considered (46–48).

### Variables Influencing Individual Responses to Creatine

#### (A) Baseline Muscle Creatine Content

The magnitude of response to creatine supplementation is typically determined by initial muscle creatine concentration (47), which can be quite variable in aging individuals and across one's lifespan. Aging adults typically have significantly lower PCr and total creatine compared to young adults (49–52), however one study reported no differences between older and younger participants (53). In contrast, Rawson et al. (54) showed that aging adults (*n* = 7) had higher resting PCr stores compared to younger adult (*n* = 8) in the gastrocnemius muscle. Chilibeck et al. (14) performed a meta-analysis to assess PCr differences between young and aging adults. Results showed that when the quadriceps, gastrocnemius, and tibialis anterior muscle groups were combined, there were no differences in muscle PCr across age groups. However, when only studies that assessed the quadriceps were included, there was an age-related reduction in intramuscular PCr, suggesting that muscle groups may respond differently.

#### (B) Muscle Fiber Type Differences

The majority of intramuscular creatine is found in type II muscle fibers (46) which progressively decreases (quantity, size) with aging [for review see Larsson et al. (8)). Syrotuik and Bell (48) showed that individuals with the highest concentration and muscle cross-sectional area of type II fibers respond more favorably to creatine supplementation. Therefore, the age-related reduction in type II muscle fiber quantity and size may potentially attenuate the anabolic response to creatine.

#### (C) Impact of Habitual Dietary Intake of Creatine

Dietary intake of creatine may also influence the responsiveness to supplementation. Individuals with low dietary consumption of creatine-containing foods [i.e., meat, seafood, poultry; (15, 19)] typically have low intramuscular creatine concentrations and would therefore respond more favorably to creatine supplementation. For example, Burke et al. (55) showed that vegetarians experienced the greatest increase in intramuscular total creatine (PCr, free Cr) from 8 weeks of creatine supplementation (0.25 g/kg lean tissue mass/day for 7 days + 0.0625 g/kg lean tissue mass/day for 49 days) compared to non-vegetarians who supplemented with creatine. Furthermore, in examining the effects of short-term creatine supplementation (0.3 g/day for 7 days) in omnivorous younger adults (*n* = 17, 29.18 ± 7.81 years) and aging individuals (*n* = 18, 71.78 ± 6.97 years), Solis et al. (52) showed that omnivores had higher dietary creatine intake pre-supplementation. Ingestion of creatine significantly increased PCr in aging individuals but not in the younger omnivore adults. There is evidence that meat consumption decreases with aging which may influence the response to creatine supplementation. We recommend that future research determine changes in intramuscular creatine concentration, type II muscle fiber quantity and size, and dietary intake of creatine so that a valid estimate regarding the effects of creatine supplementation in aging adults can be made.

#### Mechanisms by Which Creatine Supplementation Affects Muscle Mass in Aging Adults

**Figure 2:**
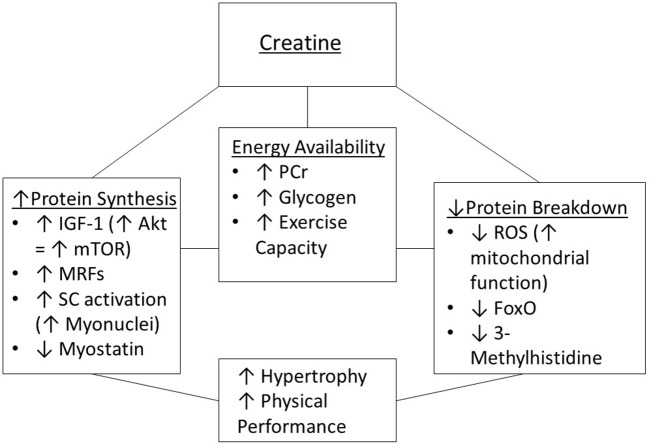
Potential mechanisms of creatine supplementation to enhance muscle hypertrophy and physical performance. IGF-1, insulin-like growth factor 1; MRFs, myogenic regulatory factors; mTOR, mammalian target of rapamycin; PCr, phosphorylcreatine; ROS, reactive oxygen species; SC, satellite cells.

Creatine supplementation may affect some of the mechanisms and pathways (those involving muscle protein synthesis and degradation) that contribute to sarcopenia (Figure 2). This section provides a review of mechanisms by which creatine may increase energy availability during resistance training, along with the effectiveness (or lack of effectiveness) of creatine supplementation for offsetting the effects of aging on muscle protein synthesis and degradation.

#### Increased Training Volume and Muscle Contraction With Creatine Supplementation Through Increased Energy Provision and Calcium Uptake Into the Sarcoplasmic Reticulum

Phosphocreatine (PCr) is important for buffering ATP levels during intense muscle contraction (i.e., resistance training). ATP, which is broken down to ADP and inorganic phosphate (Pi) during muscle contraction, can be quickly re-synthesized when PCr donates its phosphate to ADP (15). Low intramuscular PCr levels may not be able to sustain continued muscle contractions during resistance training. Aging adults have reduced intramuscular PCr levels in the upper-leg (vastus lateralis); whereas PCr levels are at normal levels in muscles of the lower-leg (gastrocnemius, tibialis anterior), those involved in low-intensity activities of daily living (i.e., walking) (14). Potentially, the reduction in PCr in the vastus lateralis may be associated with reduced participation in high-intensity physical activities (i.e., running, jumping) which involve recruitment of large muscle groups in the lower-limbs (56). There is strong evidence from studies measuring creatine and PCr levels either through muscle biopsies or ^31^P-MRS that supplementation with creatine increases muscle creatine and/or PCr levels in muscle of aging adults (33, 36, 51, 52, 54). An animal model of senescence-accelerated mice indicated that creatine supplementation may lose its effectiveness over time due to down-regulation of the creatine transporter protein in muscle; however, this was not evident in aging humans (57).

Increased intramuscular PCr may provide greater capacity for ATP resynthesis during sustained intense muscular work or may enhance PCr recovery (i.e., and therefore greater recovery between sets of resistance training exercise) because an increase of creatine in the muscle would drive recovery of PCr through the reverse of the creatine kinase reaction (i.e., ATP + Cr → PCr + ADP) (51). Furthermore, creatine increases the rate of calcium uptake into the sarcoplasmic reticulum which may increase myofibrillar cross-bridge cycling leading to shortened muscle relaxation time and greater force development (58–60). Overall, this may lead to enhanced training volume during resistance training sessions in aging adults. In a study with full exercise supervision, where sets were performed with repetitions to failure, aging males (~70 years) were able to achieve a 31% greater training volume (defined as repetitions × kg) when supplemented with creatine compared to placebo during 12 weeks of resistance training (31). This has potential to stimulate greater training adaptations (i.e., greater increases in muscle mass and strength). This beneficial effect of creatine may not hold true however if resistance training is not performed to failure, which may be the case most of the time when older adults are performing resistance training non-supervised. Furthermore, in young participants, creatine supplementation was able to maintain resistance training volume and attenuate the interference effect of concurrent training (i.e., resistance and aerobic training) on strength adaptations (61). These results may have important implications in older adults, since both resistance and aerobic training are recommended and commonly practiced for optimal health.

Aside from PCr, glycogen is an important substrate that can drive re-phosphorylation of ATP during prolonged resistance training sessions, as evidenced by significant glycogen depletion during resistance training (62). Adults (> 50 years) with type II diabetes who supplemented with creatine (5 g/day) during 12 weeks exercise training (aerobic and resistance training, 3x/week) experienced a greater increase in membrane GLUT-4 content and membrane total GLUT-4 content ratio compared to adults on placebo (40). GLUT-4 is important for transport of glucose into muscle, and since glucose is a building block for glycogen, this has potential to enhance glycogen resynthesis following resistance training. Creatine supplementation in rats for 5 days before intermittent swimming spared gastrocnemius muscle glycogen when measured post-exercise (63). Blood lactate levels during exercise were also reduced with creatine supplementation suggesting that extra energy provided by phosphoryl creatine during the intermittent exercise resulted in a smaller requirement for anaerobic glycolysis, allowing sparing of glycogen. Potentially, the sparing of muscle glycogen would allow for greater exercise training capacity and volume to be performed leading to greater muscle mass and muscle/physical performance over time.

#### Aging and Pathways for Protein Synthesis—Effects of Creatine Supplementation

Resistance training increases the release of insulin-like growth factor-1 from muscle, which may stimulate activation of proteins called “myogenic regulatory factors” [MRFs; (64, 65)]. The MRFs are involved in activation, proliferation, and differentiation of satellite cells (66), which in turn are involved in muscle fiber repair/regeneration and thought to be important in the process of muscle hypertrophy (67). Satellite cells reside outside the muscle fiber between the sarcolemma and basal membrane and when activated they fuse with the muscle fiber membrane and cross the sarcolemma where they differentiate into myonuclei (67), which increases a muscle fiber's capacity for protein synthesis (68). Myostatin is a myokine (i.e., a hormone-like protein released from muscle) that has the opposite effect to MRFs and inhibits satellite cell activation (69). There is a reduction in satellite cell number with aging (70), and a reduced activation and proliferation of satellite cells in response to a session of resistance training (71). In young muscle, MRFs are increased and myostatin is reduced following a resistance training session, which leads to activation and proliferation of satellite cells (71). These responses are attenuated with aging (71). Although there is some evidence for creatine supplementation during resistance training to increase production of IGF-1, expression of MRFs, and activation of satellite cells in younger individuals (72–74), there is no evidence of this in aging adults. Males (55–70 years) had no increase (compared to placebo) in IGF-1 when supplemented with creatine (20 g/d for 5 days + 0.1 g/kg on training days thereafter) during 12 weeks of resistance training (32). In aging males (mean age 73 years) who supplemented with creatine (5 g/day) for 7 weeks, there was no greater increase in satellite cell's or expression of MRFs in response to resistance training compared to placebo (75). Furthermore, during surgical overload of the plantaris muscle in aging rats, creatine supplementation failed to affect myonuclear domain (i.e., the amount of myonuclei for a given muscle fiber area) and did not affect muscle fiber area compared to overload without creatine (76). This indicates unchanged satellite cell differentiation.

The pathway involving phosphatidylinositol 3-kinase [PI3K]-Akt/protein kinase B [PKB]-mammalian target of rapamycin [mTOR] is important for activation of translation within muscle and is therefore important for muscle protein synthesis in response to resistance training (77). Signaling through mTOR is reduced with aging (78) but there is no direct evidence that creatine supplementation influences mTOR in aging adults.

#### Aging and Pathways for Protein Degradation—Effects of Creatine Supplementation

In contrast to studies showing a lack of direct effect from creatine supplementation on mechanisms involved in protein synthesis, creatine supplementation may be effective for reducing muscle protein degradation. In younger males, but not females, acute supplementation with creatine (i.e., 20 g/day for 5 days followed by 5 g/day for 3–4 days) reduced leucine oxidation and the rate of appearance of leucine in blood after primed continuous intravenous infusion of radio-labeled leucine, indicating reduced protein degradation (79). This was accompanied by unchanged protein synthesis in both males and females (79). A global marker of muscle protein degradation, urinary 3-methylhistidine, is reduced in aging males (but not aging females) who supplemented with creatine during resistance training (30, 41). It is proposed that during the biological process of aging, there is damage to the mitochondria, causing defects in the respiratory chain, leading to production of reactive oxygen species (80). Reactive oxygen species can cause mutations in mitochondrial DNA (encoding for respiratory chain proteins) leading to further mitochondrial damage and a vicious cycle (80). Reactive oxygen species can damage cellular membranes, leading to inflammation, muscle damage, and muscle protein degradation. Supplementation with creatine may be effective in mitigating this mitochondrial damage, leading to reduced oxidative stress, inflammation, and cellular apoptosis (i.e., cell death). In cellular studies involving human umbilical vein endothelial cells (81) or skin cells (fibroblasts) (82), or mouse myoblasts (83) exposed to oxidative damage, incubation with creatine protected against mutations in mitochondrial DNA or mitochondrial damage. It was proposed that creatine is taken up by mitochondria, where it helps maintain energy status and function by transferring phosphate groups between sites of energy production (i.e., ATP) and sites of energy consumption (i.e., to re-phosphorylate ADP) (81). There is some support for creatine having a similar effect in aging adults. Compared to a placebo group, middle-aged males (mean age 48 years) who supplemented with 20 g/day of creatine for 7 days had reduced levels of proteolytic enzymes involved in apoptosis and DNA fragmentation, and upregulation of proteins involved in protection against mitochondrial damage after a muscle-damaging exercise session [i.e., 40 min of downhill running on a treadmill; ref. (84)]. In addition, aging males (mean age of 65 years) given a daily multi-ingredient nutritional supplement (containing 2.5 g of creatine) during 12 weeks of combined resistance and high-intensity interval training, had reduced levels of inflammation (as assessed by tumor necrosis factor- alpha and interleukin-6) compared to placebo (85). In this study, the nutritional supplement also contained calcium, vitamin D, and n-3 polyunsaturated fatty acids; therefore, the effects could not be attributed solely to the creatine. Finally, in an animal model of senescence-accelerated mice, a lifetime of creatine supplementation was effective at middle age (but not oldest age) for increasing muscle carnosine content (86). Carnosine has a number of protective mechanisms within muscle including prevention of glycosylation-induced protein damage, anti-oxidant effects, and pH buffering (86). Creatine supplementation was also effective at the middle-age mark for improving muscle function [i.e., attenuating fatigue in the slow-twitch soleus, and enhancing post-fatigue force recovery in fast-twitch extensor digitorum longus; (86)]. Future longer-term studies of creatine supplementation are needed to determine if there are similar benefits in older adults.

In summary, studies determining the mechanisms by which creatine supplementation may enhance muscle accretion in aging adults favor an effect from creatine on reducing muscle protein degradation, mainly through mitigation of mitochondrial damage.

### Does the Timing of Creatine Ingestion Influence Aging Muscle?

It has been previously suggested that the strategic ingestion of creatine, in close proximity to resistance training, may help create a favorable environment for muscle growth (87). This section summarizes the limited body of research investigating the effects of the timing of creatine supplementation in response to resistance training.

In healthy aging adults who consumed creatine (0.1 g/kg) immediately before and cornstarch maltodextrin (0.1 g/kg) immediately after or cornstarch maltodextrin immediately before and creatine immediately after supervised whole-body resistance training sessions, significant improvements in upper-body (chest press) and lower-body (leg press) maximal strength were observed compared to placebo. There were no differences in strength gains between the creatine groups; however, only the group who consumed creatine post-exercise had statistically greater gains in lean tissue mass, compared to adults on placebo (37). Cribb and Hayes (88) found that a multi-ingredient supplement (including protein, carbohydrate, fat, and creatine monohydrate) in close proximity to training (i.e., immediately before and after) increased lean body mass and strength compared to ingesting the supplement in the morning and late evening (i.e., > 5 h from training) in young resistance trained participants. However, caution is warranted with a multi-ingredient study, since the impact of any individual nutrient is unknown. A small meta-analysis involving 3 studies (*n* = 80, > 18 years, ranging from 4 to 32 weeks) showed that post-exercise creatine supplementation led to greater gains in muscle mass compared to pre-exercise creatine [standardized mean difference 0.52, 95% CI 0.03–1.00, *p* = 0.04; (89)]. There were no differences between pre- and post-exercise creatine for effects on muscle strength. Loenneke et al. (90, 91) have recently provided evidence that exercise induced changes in muscle size do not contribute to exercise-induced changes in strength. Creatine has been shown to enhance training volume (24, 31), which is important for enhancing gains in muscle size, whereas training specificity seems to be more important for muscle strength (92). Although the mechanisms explaining the greater increase in muscle mass from post-exercise creatine remains to be determined, muscle contractions (during a resistance training session) stimulate creatine uptake into skeletal muscle, resulting in elevated intramuscular creatine stores (21). Greater intramuscular PCr is associated with greater muscle accretion in aging adults (36). Importantly, no study examining creatine timing has measured intramuscular creatine content, thus future research is warranted. Although the difference in muscle accumulation between pre- and post-exercise creatine is small, these results may be important for aging adults trying to maximize muscle accretion through the combination of creatine supplementation and resistance training.

### Does the Combination of Creatine With Other Nutritional Supplements Augment Muscle Mass and Performance?

Research showing a beneficial effect from creatine, in combination with other nutritional supplements, is mixed. Aging males who supplemented with creatine (*n* = 10; 67.3 ± 3.1 years; 0.1 g/kg) and whey protein (0.3 g/kg) only on resistance-training days (3 days/week for 10 weeks) experienced greater gains in lean tissue mass (5.6 ± 0.9%), as measured by air-displacement plethysmography, and upper-body (bench press) strength compared to participants on creatine (*n* = 13, 65.5 ± 2.7 years; 2.2 ± 0.8%) or placebo (*n* = 12, 64.1 ± 3.1 years; 1.0 ± 1.0%) (30). However, in frail adults (*n* = 18, ≥65 years), the combination of creatine (5 g) and whey protein (20 g) during 12 weeks of resistance training failed to produce greater gains in lean tissue mass, handgrip strength, or indices of physical performance (time-up-and-go test, timed stand test) compared to whey protein alone (38). In aging males (48–72 years), the co-ingestion of creatine (5 g) and whey protein (35 g) during 14 weeks of resistance training increased whole-body lean tissue mass and measures of whole-body strength similarly to that of creatine or protein alone (28). Furthermore, Villanueva et al. (34) found no differences in muscle accretion, bench press strength, stair climbing power, or 400-m walk time from the combination of creatine (0.3 g/kg for 5 days + 0.07 g/kg for 68 days) and whey protein (35 g/day) during 12 weeks of resistance training compared to resistance training alone in aging males (68.1 ± 6.1 years). Results across individual studies suggest that the combination of creatine and protein does not provide additional muscle benefits compared to creatine or protein alone in aging adults.

The ingestion of a multi-ingredient supplement containing creatine (5 g), whey protein (60 g), vitamin D (1000 IU), EPA (2,800 mg), and DHA (1,780 mg) for 6 weeks significantly increased whole-body lean tissue mass (assessed by DXA), and upper-body strength in aging males (*n* = 25, 71 ± 1 years) compared to aging males on placebo (*n* = 24, 74 ± 1 years) (93). Interestingly, continued ingestion of the supplement during 12 weeks of supervised aerobic (1x/week) and resistance training (2x/week) did not lead to greater gains in lean tissue mass. Aging adults (*n* = 21, 65–85 years) who supplemented with creatine (5 g) and conjugated linoleic acid (CLA; 2 g) daily during 6 months of resistance training experienced greater gains in muscle accretion (2.1 kg) compared to those on placebo (*n* = 18; 0.9 kg increase) (43). The combination of creatine and CLA also increased lower-limb isokinetic strength and total-body muscle endurance (chest press, arm flexion, knee extension; females only). While it is difficult to determine whether creatine is responsible for the greater gains in muscle mass across individuals studies, a recent systematic review and meta-analysis performed by O'Bryan et al. (94) concluded that multi-ingredient supplements containing protein and creatine resulted in significantly greater gains in muscle mass compared to protein alone (1.01 kg; 95% CI [0.69, 1.33], *p* < 0.00001). Furthermore, Chilibeck et al. (14) showed that creatine supplementation during resistance training resulted in superior gains in muscle mass compared to placebo, even when studies involving protein and CLA were excluded from the meta-analysis.

### Safety of Creatine Supplementation-Aging Adults and Clinical Populations

There is limited research regarding the safety profile of creatine supplementation. Self-reported adverse effects in aging adults from creatine include muscle cramping, muscle strains and gastrointestinal irritation. Using a retrospective questionnaire, Chrusch et al. (31) reported that 12 weeks of creatine supplementation (0.3 g/kg/day × 5 days + 0.07 g/kg/day thereafter) in aging males increased the incidence of muscle pulls and muscle strains compared to placebo. In aging adults (*n* = 11) who consumed creatine (0.3 g/kg) for 10 days, four adverse events were reports [pulled groin muscle, gastrointestinal irritation, constipation, bloating; (95)]. In aging postmenopausal women who ingested creatine (0.1 g/kg/day) during 52 weeks of resistance training, five participants reported symptoms of gastrointestinal irritation and two reported muscle cramping, which was higher (*p* < 0.05) than the adverse effects reported by females on placebo (25). However, several other studies indicate no adverse effects (self-reported) from creatine supplementation (23, 29, 37, 38).

There is no direct evidence that creatine supplementation causes cytotoxicity (urinary formaldehyde) in aging males (30) or has an adverse effect on kidney or liver function. Creatine supplementation (5 g/day) during 14 weeks of resistance training in frail adults resulted in no detrimental effect on kidney or liver function (38). In two studies involving aging post-menopausal females, creatine supplementation, with and without resistance training, had no effect on urinary albumin (96) or other markers of kidney (urea, microalbumin, urine protein, creatinine clearance) or liver function (bilirubin, aspartate aminotransferase, alanine aminotransferase, and alkaline phosphatase) (25). The co-ingestion of creatine and CLA during 6 months of resistance training had no effect on bilirubin or gamma glutamyltransferase protein in aging adults (43). From a clinical perspective, creatine supplementation did not alter kidney function (renal damage, hematuria, tubular damage, glomerular filtration rate, microalbumin) compared to placebo in aging patients with Parkinson Disease (97) or affect albuminuria, proteinuria, albumin: creatinine ratio, urea and creatinine and estimated creatinine clearance in aging adults with type II diabetes (40). In summary, self-reported adverse events from creatine supplementation include gastrointestinal issues and muscle pulls/strains. Direct assessment of kidney and liver function indicates no negative effect from creatine in aging adults.

## Conclusions

Sarcopenia is an age-related muscle condition characterized by a reduction in muscle quantity, muscle performance (i.e., strength) and physical performance (i.e., tasks of functionality). Although multifactorial, sarcopenia may be caused by changes in muscle protein kinetics, neuromuscular function, inflammation, physical activity, and nutrition. Recent attention has focused on nutritional interventions as a potential therapeutic approach to counteract sarcopenia. When consumed during resistance training, creatine supplementation typically increases muscle mass and muscle/physical performance, possibly by influencing high-energy phosphate metabolism and calcium uptake, muscle protein kinetics, and inflammation. However, variability in the responsiveness to creatine supplementation is typically high in aging adults and factors such as initial intramuscular PCr concentration, type II muscle fiber content and size, and habitual dietary intake of creatine may possibly explain the inconsistent findings across individual studies. Furthermore, methodological issues such as sex, exercise training intervention, sample size, and participant health status may also influence study results.

## Author Contributions

DC, SF, PC, SC, JA, and RK contributed to the design, methodology, evaluation, writing, edits, and approval of the submission.

### Conflict of Interest Statement

The authors declare that the research was conducted in the absence of any commercial or financial relationships that could be construed as a potential conflict of interest.
